# Multi-centre randomised controlled feasibility trial with embedded process evaluation of a samba percussion intervention for people living with Parkinson’s disease: a protocol for the Sparky Samba trial

**DOI:** 10.1136/bmjopen-2025-114372

**Published:** 2026-09-09

**Authors:** Rebecca Hamilton, Eirwen Malin, Nigel Kirby, Rhys Williams-Thomas, Philip Pallmann, Kim Smallman, Duncan Mclauchlan, Nina Jacob, Jazmine Baxter, Chris Jones, Claudia Metzler-Baddeley, Katy Hamana, Cheney JG Drew

**Affiliations:** 1 Centre for Trials Research, Cardiff University, Cardiff, UK; 2 Public Contributor, Cardiff, UK; 3 Department of Neurology, University Hospital Wales, Cardiff, UK; 4 School of Psychology, Cardiff University, Cardiff, UK; 5 School of Healthcare Sciences, Cardiff University, Cardiff, UK

**Keywords:** Parkinson-s disease, Community Participation, Clinical Trial

## Abstract

**Introduction:**

Parkinson’s disease (PD) is the second most common neurodegenerative disorder, its principal symptom being deterioration of motor function. Current treatment options are limited to symptom management but there is evidence that physical activity can provide motor benefits. More recently there is evidence to suggest that rhythmic auditory stimulation may improve gait and balance in PD. Sparky Samba is a community initiative in South Wales, UK, founded by a person living with PD. Sessions incorporate the following samba rhythms from a trained facilitator and are held weekly in a community setting.

**Methods:**

The Sparky Samba trial is a multi-site, non-blinded, randomised controlled feasibility trial of Sparky Samba compared with activity as usual. A total of 60 people with PD will be randomised 1:1 to take part in a local Sparky Samba group for 12 weeks or continue their normal activities for the same length of time. The primary outcome is feasibility defined by recruitment, retention, data completeness and intervention adherence. Secondary outcomes relating to motor function, cognition, well-being and self-efficacy will also be assessed at baseline and at 12 weeks. Additionally, we will conduct a process evaluation to understand contextual mechanisms surrounding Sparky Samba. This will be achieved through qualitative interviews and structured participant questionnaires following individual trial completion and through structured questionnaires with intervention delivery staff, supplemented with qualitative interviews.

**Analysis:**

Feasibility outcomes will be assessed according to pre-defined criteria. For secondary outcomes, means and standard deviations (or medians and IQRs) will be calculated by arm, alongside 95% CIs for change from baseline to 12-week follow-up. Qualitative data will be subject to thematic analysis using NVivo software.

**Ethics and dissemination:**

This study received a favourable ethical opinion from the North of Scotland Research Ethics Committee in April 2025 (REC reference 25/NS/0037). Study results will be disseminated through the peer-review literature, the ISRCTN registry and directly to participants, which will be facilitated by the study’s public and patient involvement steering group.

**Trial registration number:**

ISRCTN11861663

**STRENGTHS AND LIMITATIONS OF THIS STUDY**

- This feasibility study originated from a person living with Parkinson’s disease.
- The trial design has included significant co-development with public and patient representatives.
- Inclusion of the process evaluation provides granular assessment of operational feasibility.
- The comparator condition (usual daily activities) will be highly variable between individuals.
- The activities as usual comparator does not fully control for the social interaction component of Sparky Samba.

## Introduction

 Parkinson’s disease (PD) is the second most common neurodegenerative disorder after Alzheimer’s disease, causing cell loss in the brain largely due to the abnormal aggregation of the alpha-synuclein protein. In PD, atrophy of dopaminergic cells in the substantia nigra leads to progressive decline of motor function alongside changes in cognition, sleep, mood and other non-motor symptoms. There are no disease-modifying therapies, with pharmacological treatment options primarily supporting motor symptom management and often with significant side effects that worsen other PD symptoms. Stakeholder research prioritisation exercises demonstrate the need for developing non-pharmacological interventions that may aid both motor and cognitive functions^1 2^ and allow tailoring for disease stage.^3^

Common non-pharmacological interventions shown to have both motor and non-motor benefits for people with PD (PwP) include physical activity in the form of targeted exercise^4 5^ or dancing^6^ and neurological music therapy techniques such as rhythmic auditory stimulation (RAS).^7^ RAS involves the synchronisation of walking pace to external rhythmic beats and has shown promising effects on gait and mobility in PwP.^8–10^ The majority of these interventions have been trialled in clinical settings, but it is recognised that community-based activities promoting healthy behaviours in PwP are likely to have greater long-term impacts^11–13^ due to their enhanced accessibility and effects of social cohesion.

A recently established PD-specific samba percussion group in South Wales, UK, called ‘Sparky Samba’, is a patient initiative designed and led by PwP for PwP and supported by a samba percussion tutor. This group’s activity combines rhythmic upper and lower limb activities with social stimulation. Awareness of Sparky Samba has gained momentum among PwP who have indicated a need for similar groups in other local communities across the region. An initial qualitative evaluation of Sparky Samba, conducted by our group (unpublished data), has highlighted its utility as a community-based activity for PwP with the potential to improve social connectedness, well-being, physical activity participation and cognition. As indicated by our trial research partner (the founder of Sparky Samba), PwP have expressed a preference for therapeutic activities and lifestyle interventions that they themselves can control. Patient-led research is essential to develop interventions that have a better chance of successfully translating into practice because they are inherently acceptable, meaningful and relevant to those they have been developed by and for.^14–16^

We have conducted an ethnographic study of the existing Sparky Samba group in Cardiff, with follow-up stakeholder workshops in order to: (1) identify core intervention components, including duration, type of exercises, movements, resources, delivery and specific content in preparation for intervention delivery; (2) refine intervention components and determine intervention flexibility and how this may be adapted for individuals; (3) describe and specify the resulting intervention in detail according to the TIDieR framework^17^ and (4) create a logic model and identify a ‘theory of change’ regarding intervention mechanisms (how it may work) and identify what may impact those mechanisms of change.

Sparky Samba has achieved support from the PD community (both PwP and the UK’s leading PD charity, Parkinson’s UK (PUK)) and arts-based funders but it warrants further investigation for its potential health benefits. The Sparky Samba intervention will be compared with an activities as usual control group. Our PD community members have indicated many people take part in other PD-specific groups, such as seated bowling or choirs, which have a similar duration to Sparky Samba sessions but are sporadic in their geographic availability. By using an activities-as-usual control, we aim to differentiate between the community and social aspects of standard social activities versus the specifics of the Sparky Samba intervention and potential benefits of rhythmic-based activities.

The aim of this study is to evaluate the feasibility of Sparky Samba in a small-scale randomised controlled trial (RCT) as a next step towards a full effectiveness evaluation of Sparky Samba as a health intervention for PwP.

## Methods and analysis

### Public and Patient involvement

The Sparky Samba groups were founded by a person living with PD. The initiative commenced with a single group in South Wales, which has now expanded to three groups across Wales. As a result of the benefits that initial group members perceived from participation, they sought to engage with robust scientific evaluation, forming a collaboration with academic and clinical researchers to develop this trial. Subsequently, the original Sparky Samba group have remained engaged throughout the process of developing and delivering this research. Therefore, not only is the Sparky Samba trial driven by the patient voice, but it is an integral factor in its ongoing delivery. The founder of Sparky Samba aided in securing funding for the trial and continues to engage through regular trial management group (TMG) meetings, along with an additional person who lives with PD. Additionally, the original Sparky Samba group members attended a series of three workshops to discuss and refine trial design, help with the selection of outcome measures and aid in defining the parameters for intervention adherence. They have also been involved in the development of patient-facing materials as well as intervention refinement. At the conclusion of the trial, we will work with this group on generating resources to communicate the trial results to the PD community alongside the development of a patient-focused dissemination strategy.

### Trial design and setting

The Sparky Samba trial is a parallel-group feasibility RCT of the Sparky Samba intervention compared with an activities-as-usual control group. We will recruit 60 PwP to be randomised 1:1 to the intervention or activities as usual for a period of 12 weeks. The recruitment target of 60 has been selected in line with guidance for sample sizes in feasibility studies.^18^ This sample size will allow estimation of recruitment, retention, adherence and data completeness rates with a 95% binomial CI of no more than plus or minus 13 percentage points irrespective of the point estimate. The trial is not powered for hypothesis tests to detect effects in the clinical outcome measures.

The trial will be conducted in three secondary care (National Health Service (NHS)) sites across Wales, UK, with the Sparky Samba intervention delivered at three local community sites identified and supported by the Sparky Samba group. The clinical sites have been selected due to their co-location with the Sparky Samba groups to facilitate attendance of participants at the Samba groups.

### Objectives

The primary objective of the Sparky Samba trial is to determine the feasibility of conducting a full-scale effectiveness RCT of the Sparky Samba intervention. The secondary objective is to explore the effect of Sparky Samba on functional and well-being measures, compared with activities as usual, in PwP.

### Recruitment and consent

Potential participants will be identified through specialist secondary care provisions (including both movement disorder and care of the elderly clinics) by clinical staff (doctors, nurses and PD specialist nurses). The trial will also be advertised on social media networks and through Parkinson’s UK Cymru, permitting participants to self-refer directly to the trial team. A screening log will be kept at each site and with the central trial team for analysis of feasibility measures pertaining to recruitment.

Participants are eligible for the trial if they meet all of the following inclusion criteria and none of the exclusion criteria, which have been defined to enable broad inclusivity of PwP. The inclusion criteria are: (i) anyone diagnosed with PD, (ii) aged 18 or older and (iii) able to provide informed consent. The sole exclusion criterion is previous and/or present participation (self-reported) in a Sparky Samba group (this does not exclude people with prior experience of music or dance).

Interested participants will be provided with a detailed information sheet providing information on the trial and what participation entails. Following confirmation of eligibility (either by clinician or self-report), participants will be registered on the trial database, constructed in Research Electronic Data Capture (REDCap),^19 20^ where they are automatically assigned a unique identification number. This number is used to label all study data to ensure participant confidentiality. Registration requires the provision of contact details to enable distribution of online patient-reported outcome measures, and consent is explicitly sought for the storage of this data. Access to contact details is restricted to the central trial team via password access to REDCap. Once registration is complete, participants are then asked to complete the online consent form in REDCap. Participants may also choose to complete the consent form on paper. If this option is chosen, completed consent forms will be stored at the research site.

Participants may withdraw their consent to participate at any time without their rights or clinical care being affected. Within the initial study information, it is made clear to participants that they can withdraw from all or specific parts of the trial (intervention, follow-up questionnaires, etc.) and that at the point a decision to withdraw is made, de-identified data collected about them will be retained for analysis.

### Randomisation

Participants will be randomised to the intervention or activities as usual in a 1:1 ratio, stratified by disease burden using the Hoehn and Yahr score of disease stage.^21^ The randomisation sequence, generated by the non-blinded, senior trial statistician, using nQuery software and stored securely so those enrolling participants cannot access it, will use permuted blocks of randomly varying sizes. The randomisation will be implemented via REDCap, and allocation to groups will be performed by the central trial team. Allocation will be communicated directly to the participant with concurrent notification to the recruiting site and, if required, the relevant local Sparky Samba group. Randomisation should occur within 14 days of the baseline assessment.

Owing to the nature of the intervention, this is a non-blinded trial. However, the trial statistician will remain blind to allocation until analysis is complete. Further, the Unified Parkinson’s Disease Rating Scale Part III^22^ will be video recorded, and ratings will be performed by a trained clinician blind to the allocation of the participant.

### Intervention

Sparky Samba is a samba band-based rhythm/drumming intervention delivered within the community for PwP. The setting is determined by the local availability of facilities, which are chosen by Sparky Samba leaders based on their geographic location and accessibility for group members. The group is facilitated by either a local samba musician or a regular member of the group who has been trained by the musician to deliver Sparky Samba sessions. The group size is usually around 8–15 participants but there is no minimum or maximum number required for any given session. While the sessions are designed specifically for PwP, supporters of PwP or partners of PwP are welcome to engage in the group sessions if they so wish.

**Figure 1:**
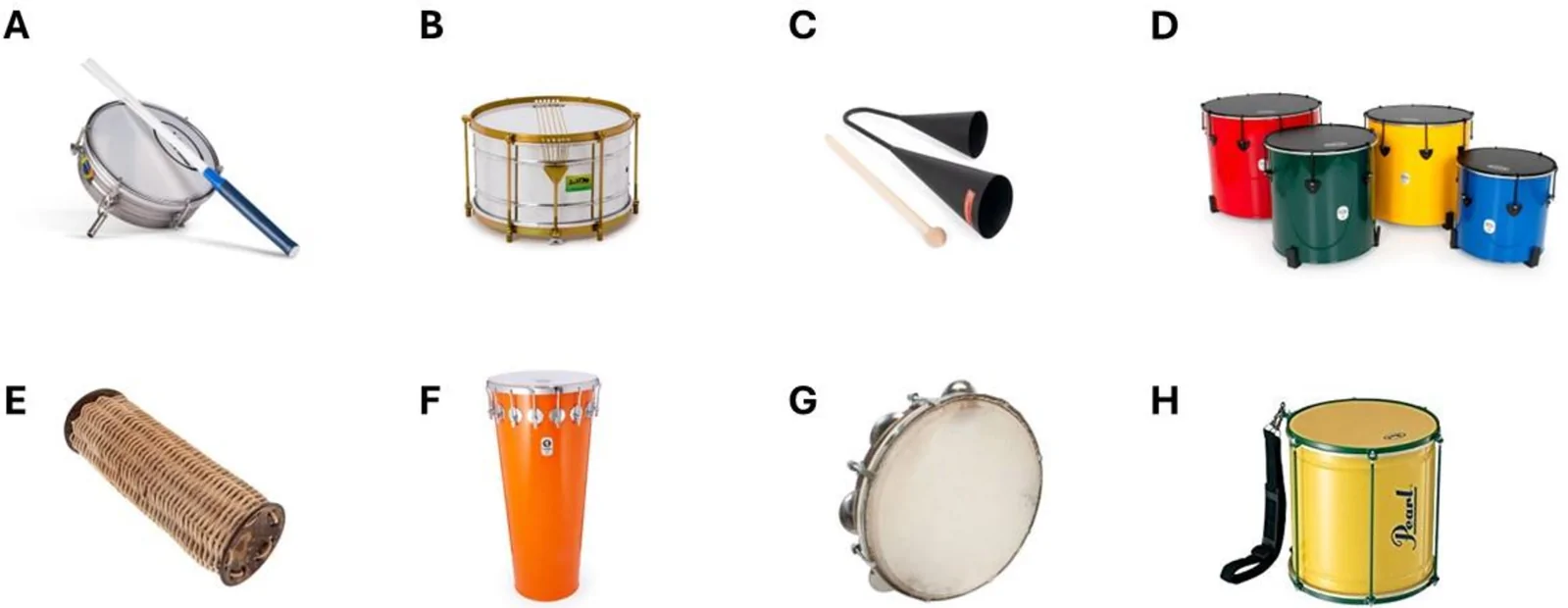
Representative images of the range of instruments that may be used in a Sparky Samba session. A – Tamborim, B - Caixa, C – Agogo bells, D – Surdos, E – Ganzá, F - Timbal, G – Pandeiro and H - Repinique.

In their first session, participants will be offered a choice of instrument that is suitable for their range of motion. This could range across tamborims, Snare drums (Caixa), Agogo bells, Surdos, Ganzá/Chocalho (shakers), Cuíca, Timbal, Pandeiro and the Repinique (figure 1). The Repinique, Timbal and Surdo drums are usually played in a standing position, whereas the Tamborim, smaller Surdo drums, Caixa, Agogo bells, Ganzá/Chocalho and Pandeiro can be played in a seated position. The Repinique is carried by the player using a strap over the shoulder which suspends the drum just below the drummer’s waist. Most instruments require at least one stick to play except the Ganzá/Chocalho or Pandeiro, which require movement of only one upper limb to play. During each session, participants will be instructed to follow a rhythmic sequence, as guided by the facilitator. Typically, the room is arranged with participants in a semi-circle, grouped by instrument type, facing the facilitator. Each rhythm is broken down into smaller sections with sub-groups (grouped by instrument) concentrating on their own section before coming together. The band leader/facilitator often carries a Repinique, as well as using Apitos (whistle) to signal breaks to participants. Each session lasts for approximately 60 min with time on either side of the session for the set-up and breakdown of instruments and seating.

Work aligned to this trial has involved determination of the key factors constituting Sparky Samba as a health intervention which will be published elsewhere (manuscript under review).

Participants will be asked to attend as many sessions as possible, ideally weekly over a period of 12 weeks following randomisation. The Public and Patient Involvement (PPI) group stressed the importance of providing adequate notice to participants prior to starting the intervention, as individuals may need time to make necessary arrangements. Therefore, participants are given a 2-week grace period following randomisation to complete their first intervention session. Participants will be asked to attend at least six consecutive sessions and a minimum of 8 in total (completion of eight sessions in total will be regarded as adherent to the intervention). Adherence will be recorded by both self-report questionnaires and attendance registers from the Sparky Samba groups. The costs of participant travel to attend Sparky Samba sessions will be reimbursed. Alternatively, travel arrangements will be made upfront on behalf of the participants if they prefer. Following completion of the 12-week intervention period, participants can continue to attend the Sparky Samba group.

### Activities as usual

Participants randomised to the activities as usual control group will be asked to continue with their normal activities and attend any social or support groups they would ordinarily participate in over the 12 weeks. Participation in the trial does not necessitate prior or ongoing attendance at a particular social or support group. A physiotherapy or physical activity control group was considered to control for the type of movement included in a Sparky Samba session. However, this was discounted because while there is good evidence that physiotherapy and exercise can benefit PwP,^23 24^ the delivery of physiotherapy and exercise to this patient group, either through healthcare systems or community groups, is inconsistent across the region. Further, in conjunction with our PPI group, we were conscious that the opportunity for social interaction was a key aspect of attending Sparky Samba, which would not be achieved through attendance at healthcare-delivered physiotherapy sessions. The PPI group also suggested that many PwP are involved in other local community groups; therefore, activities as usual was chosen as the control. From the input of the PPI group, we anticipate that activities as usual may consist of activities such as walking, the gym, seated bowls or less physically orientated activities such as a Parkinson’s choir. Given the varied availability of specific Parkinson’s groups or organised community groups, we have not specified activities as usual but will be asking people randomised to this group to specify the activities they have taken part in over the 12-week trial period. This will help us determine the feasibility of using ‘activities as usual’ as a comparator condition in a future effectiveness trial of Sparky Samba. Following completion of the 12-week follow-up assessment, participants randomised to activities as usual will be offered the opportunity to join their local Sparky Samba group. As for trial participants initially randomised to the intervention group, any costs associated with attendance at the Sparky Samba group will be supported by the trial for a period of 12 weeks.

During the intervention delivery period, there are no restrictions imposed on any additional treatment participants may require for their PD or otherwise. If changes in health status and/or treatment affect their participation in the trial, this will be recorded via notification of withdrawal.

### Outcomes

The primary outcome for this trial is feasibility as defined by participant recruitment, retention, intervention adherence and data completeness according to pre-specified criteria. Thresholds for the feasibility criteria are detailed in table 1.

**Table 1:** Measures of feasibility and pre-defined criteria for success used to determine the primary outcome

Variable	Progression criteria		
	Red	Amber	Green
Recruitment			
% of people approached who are willing to participate (100*(number consented/number approached)%)	<5%	5–19.9%	≥20%
Number recruited	<25	25-44	45-60
Retention			
% of participants attending the primary 12-week endpoint assessment (100*(number attending/number consented)%)	<50%	50–89.9%	≥90%
Adherence			
% of participants adherent to the intervention (attending ≥8 sessions) (100*(number adherent/number consented)%)	<50%	50–79.9%	≥80%
Data completeness			
% of participants completing at least 90% of baseline measures (100*(number completing ≥90%/number consented)%)	<70%	70–94.9%	≥95%
% of participants completing at least 90% of follow-up measures (100*(number completing ≥90%/number consented)%)	<50%	50–74.9%	≥75%

Secondary outcomes to be collected include those measuring motor function, cognition, well-being, participation and self-efficacy. Individual outcomes are detailed in table 2. Functional outcomes are assessed within the hospital setting at baseline and again at 12 weeks following the intervention delivery period (table 3). Site staff delegated to perform the functional assessments will receive training from the central trial team at the site initiation visit, with an accompanying assessment manual and ad-hoc support from the trial team and clinically qualified members of the TMG. Demographic data (age, sex, ethnicity and educational level) and current medications will also be collected at baseline to provide cohort descriptives. Intervention adherence will be recorded weekly through self-report by the participant and by report from the Sparky Samba group leader/local organiser. At the end of the 12-week trial period, participants in the control group will be asked to detail the activities they have participated in for the previous 12 weeks. As retention is part of the feasibility outcome and the follow-up period is just 12 weeks long, we will not be enacting any specific strategies to boost retention beyond supporting travel to intervention sessions and enabling the flexibility described for completing outcome assessments.

**Table 2:** Secondary outcomes collected in the Sparky Samba trial

Construct	Measure
Motor	Unified Parkinson’s Disease Rating Scale (UPDRS) section III(^22^): we will use this to assess global motor function in participants.
	Freezing of Gait (FoG)(^28^): this is a patient self-report measure designed to assess the severity of gait freezing in PwP.
	Dresden Falls Questionnaire (DREFAQ)(^29^): this is a patient self-report questionnaire designed to assess the risk and severity of falls, a common symptom in PwP.
	Mini-Balance Evaluation Systems Test (Mini-BES Test)(^30^): in this assessment participants are assessed across dynamic balance, functional ability and gait.
	Physical Activity Scale for the Elderly (PASE) (^31^): this questionnaire is designed to capture the level of physical activity across a range of usual activities over the past 7 days.
Cognition	Montreal Cognitive Assessment (MoCA)(^32^): we will use the truncated on-line version of the MoCA, known as XpressO, to assess cognitive function in participants. This is well validated for assessing cognitive function in PwP.
Quality of Life	Parkinson’s Disease Questionnaire (eight items) (PDQ-8)(^33^): this is a self-completion participant-reported outcome designed to address aspects of functioning and well-being for those affected by PD. The PDQ-8 asks one question from each of the domains of the longer PDQ-39; activities of daily living, mobility, emotional well-being, stigma, social support, cognition, communication, bodily discomfort, with questions about thoughts of death and self-harm being removed.
Self-Efficacy	Oxford Participation and Activities Questionnaire (OxPAQ)(^34^): this is a validated self-report questionnaire designed to assess the degree of activity and participation of those with chronic conditions taking part in community-based interventions.
	Lorig Self-Efficacy Scale(^35 36^): this scale will be used to measure self-efficacy related to exercise (exercise sub-scale only).

PD, Parkinson’s disease; PwP, people with PD.

**Table 3:** Participant timeline for completion of trial assessments and intervention delivery

Procedures	Visits					
	Baseline visit (week 0)			Intervention delivery (days 0–84+14 days)	Follow-up visit (week 12±5 days)	
	At home (days −3±2 days)	At hospital (day 0)	Remote (days 0+14 days)		At home (days 85±5 days)	At hospital (days 88±2 days)
Informed consent	x					
Demographics	x					
Medications		x				
Hoehn and Yahr staging		x				
Randomisation			x			
Intervention delivery				x		
Intervention adherence				x	x	
UPDRS		x				x
FoG	x				x	
Mini-BES Test		x				x
DREFAQ	x				x	
MoCA (XpressO)		x				x
PDQ-8	x				x	
OxPAQ	x				x	
Lorig	x				x	
PASE	x				x	
Adverse event assessments				x	x	x
Process evaluation questionnaire					x	

DREFAQ, Dresden Falls Questionnaire; FoG, Freezing of Gait; Mini-BES Test, Mini-Balance Evaluation Systems Test; MoCA, Montreal Cognitive Assessment; OxPAQ, Oxford Participation and Activities Questionnaire; PASE, Physical Activity Scale for the Elderly; PDQ-8, Parkinson’s Disease Questionnaire (eight items); UPDRS, Unified Parkinson’s Disease Rating Scale.

### Safety monitoring

Adverse events will be recorded at the 12-week follow-up assessment, and participants will be encouraged to self-report any serious adverse events during the intervention delivery period to their recruiting site for inward reporting. Falls are a known facet of living with PD and may be regarded by some participants as an everyday occurrence. However, we will attempt to record all incidences of falls regardless of severity.

### Process evaluation

To understand barriers and facilitators to intervention delivery as well as the contextual mechanisms for potential effectiveness, we will embed a process evaluation in line with Medical Research Council guidance for the evaluation of complex interventions.^25^ The process evaluation will use both qualitative and quantitative data. All intervention delivery staff will be asked to take part in semi-structured interviews, conducted by a trained qualitative researcher. Additionally, intervention delivery staff will be asked to contribute reflections on intervention delivery, including specific items pertaining to fidelity of intervention delivery, in a structured questionnaire. This will be used to assess fidelity and understand the key mechanisms of change. Trial participants and their support partners (if willing) will be asked to complete a structured questionnaire that focuses on their views of the trial and of the intervention using a mix of Likert-style questions and free text responses. Participants can choose to type the free text responses or respond to these in a semi-structured interview. We will also attempt to contact any participants who drop out of the intervention to ascertain reasons for discontinuing.

### Data collection and management

Study data will be collected and managed using REDCap electronic data capture tools hosted at Cardiff University.^19 20^ REDCap is a secure, web-based software platform designed to support data capture for research studies, providing (1) an intuitive interface for validated data capture, (2) audit trails for tracking data manipulation and export procedures, (3) automated export procedures for seamless data downloads to common statistical packages and (4) procedures for data integration and interoperability with external sources.

All trial data recorded in REDCap features in-built data validations and mandatory completion of specific variables to promote quality control and data completion. Outcome data will be collected through a combination of patient-reported outcome measures (PROMs) completed directly within the database and in-person assessments conducted by research staff at the site (table 3). Following the provision of informed consent and confirmation of a baseline appointment from trial staff at the recruitment site, participants will be emailed a link to complete the PROMs ahead of their baseline assessment. If a participant chooses, they can complete the PROMs on paper copies at their baseline appointment. If a participant attends their baseline or follow-up appointment without having previously completed the PROMs in REDCap, they will be supported to do so either face to face at the appointment or by telephone by a member of the trial team.

Intervention adherence data will be collected directly from participants via REDCap. Participants will be emailed a brief attendance checklist weekly for up to a period of 14 weeks from the point of randomisation. Samba group leaders and group coordinators (local volunteers) will be asked to provide data on attendance on a pre-populated register provided to them by the central trial team.

Similarly, process evaluation questionnaires will be distributed through REDCap for remote completion. Process evaluation interviews will be conducted remotely using secure video conferencing software. Interviews will be audio recorded for verbatim transcription. Audio recordings will be deleted once transcripts have been cleaned, de-identified and incongruences verified. De-identified transcripts will be stored on a restricted access computer drive at Cardiff University.

All data management processes for the trial are contained in the Sparky Samba Data Management Plan.

### Data monitoring

A monitoring plan has been developed according to the trial risk assessment. Given the low-risk nature of the study, no routine on-site monitoring is planned. Consent and participant eligibility provided via REDCap is monitored following notification of participant registration. Outcome data entered into REDCap is monitored for accuracy and spurious entries on a monthly basis and any queries raised with sites via e-mail. All data queries, participant status metrics (recruitment, withdrawal, data completeness and intervention adherence), safety events and protocol non-compliances are monitored monthly at TMG meetings.

### Analysis

Key demographic data will be summarised using descriptive statistics. For feasibility outcomes, recruitment will be assessed as the percentage of people approached who provide consent to participate in the trial; retention as the percentage of participants recruited who attend the 12-week follow-up assessment (primary endpoint); adherence as the percentage of participants adherent to the intervention (where intervention adherence is defined as attending ≥8 of 12 sessions) and data completeness as the percentage of participants completing ≥90% of baseline and follow-up measures, respectively (table 1). Feasibility criteria will be assessed according to a traffic light system with green (proceed to full effectiveness RCT) if all criteria listed in table 1 are in the green zone; amber (requiring adjustment before proceeding to full RCT) if all criteria are either in the amber or green zone; and red (do not progress to full RCT unless major changes are implemented) if at least one criterion is in the red zone. For secondary outcome measures, we will calculate descriptive summaries by arm, such as mean and standard deviation (or median and IQR, in case of a distinctly non-normal distribution) and 95% CIs of participants’ changes from baseline to 12-week follow-up. This will provide estimates of effect size and variability of drumming-induced changes for use in power calculations for a future large-scale RCT. No sub-group, sensitivity or interim analyses will be conducted. Reporting of results will follow the Consolidated Standards of Reporting Trials (CONSORT) guideline extension for pilot and feasibility studies.^26^ The statistical analysis plan will be available via ISRCTN (*ISRCTN11861663*).

Following each interview, notes will be re-read and audio recordings replayed to identify initial ideas or areas of interest. Interviews will be transcribed verbatim, and transcripts along with free text responses to the questionnaires will be analysed thematically.^27^ Analysis will be an iterative and ongoing process throughout data collection. After familiarisation with the data, preliminary codes will be generated to label data relevant to the research objectives. Coded data will then be collated to generate themes, with summaries produced for each theme to map intervention components and activities, contributing to the development of a logic model. We will visually arrange the data to build an overall picture of the whole data set. The next stage will involve the research team using the summaries to examine the quality and boundaries of themes identified. From this, we will finalise a thematic map, refining the specifics of each theme to capture key concepts and produce analytical commentary and interpretation of the data set as a whole and connect it with the original research objectives. The qualitative software package, NVivo (2015), will be used to manage the data. The data and analysis will be reviewed by members of the research team and checked for accuracy with those taking part in Sparky Samba.

### Ethics and dissemination

A favourable ethical opinion for the trial was received from the North of Scotland Research Ethics Committee on 14 April 2025 (REC reference 25/NS/0037) followed by approval from the Health Research Authority (HRA) and Health and Care Research Wales (HCRW) on 25 April 2025. A substantial amendment to the project, which included the addition of a further patient-facing document to be given to participants randomised to the intervention arm, providing local Sparky Samba group details, was approved on 1 July 2025. Any future amendments to the trial protocol will be communicated to individual sites following REC and HRA/HCRW approval. The trial protocol is available via ISRCTN (ISRCTN11861663.)

Results of the trial will be published in the peer-reviewed literature and alongside the trial registration at ISRCTN. A plain language summary of the results will be made available at https://www.cardiff.ac.uk/centre-for-trials-research/research/studies-and-trials/view/Sparky-samba and will also be shared through patient advocacy organisations such as PUK. Additionally, results of the study will be communicated to all participating sites and study participants directly. The design and content of outputs and any additional dissemination will be informed by our PPI steering group.

## Data Availability

Data sharing not applicable as no datasets generated and/or analysed for this study.
