# Supplementary material for: Multi-centre randomised controlled feasibility trial with embedded process evaluation of a samba percussion intervention for people living with Parkinson’s disease: a protocol for the Sparky Samba trial
Source: BMJ Open. 2026 Sep 9;16(9):e114372. doi: 10.1136/bmjopen-2025-114372 (PMC13560982; doi:10.1136/bmjopen-2025-114372)
Supplement: Supplementary file 1 [file bmjopen-2025-114372.reviewer_comments.pdf]

PEER REVIEW HISTORY

BMJ Open publishes all reviews undertaken for accepted manuscripts. Reviewers are asked to complete a checklist review form and are provided with free text boxes to elaborate on their assessment. These free text comments are reproduced below.

ARTICLE DETAILS

Title (Provisional)

A multi-centre randomised controlled feasibility trial with embedded process evaluation of a samba percussion intervention for people living with Parkinson's disease: a protocol for the Sparky Samba trial

Authors

Hamilton, Rebecca; Malin, Eirwen; Kirby, Nigel; Williams-Thomas, Rhys; Pallmann, Philip; Smallman, Kim; Mclauchlan, Duncan; Jacob, Nina; Baxter, Jazmine; Jones, Chris; Metzler-Baddeley, Claudia; Hamana, Katy; Drew, Cheney JG

VERSION 1 - REVIEW

**Reviewer**	**1**
**Name**	**Ni Bhriain, Orfhlaith**
**Affiliation**	**University of Limerick**
**Date**	**07-Jul-2026**
**COI**	**None**

Very thorough, well constructed paper. I appreciate the extent to which it is championed by People with PD and welcome the co-design element.

Has consideration been given to participants with hearing devices?

Are partners, carers welcome to attend the sessions?

Will previous music/ dance experience of participants be considered?

P 23 Typo LINE 15 lot/Lost

P 37 Typo line 36 Participants

**Reviewer**	**2**
**Name**	**Urrea Mendoza, Enrique**
**Affiliation**	**Florida State University**
**Date**	**12-Jul-2026**

**COI**	**None**

Thank you for the opportunity to review the paper entitled “A multi-centre randomize controlled feasibility trial with embedded process evaluation of a Samba percussion intervention for people living with Parkinson’s disease: a protocol for the Sparky Samba trial”. This is an interesting topic and the manuscript is updated and well writing. However, in order to consider publication I want clarification about enrollment and the intervention dates. It seems like the final enrollment was done on February 2026 and at this time the researchers should be in the phase of analyzing data. Per guidelines this journal can’t publish protocols if data collection is completed.

Additionally, because this is a new intervention, a better description of the methodology, including description of instruments and graphics, will be useful for the readers that are not familiar with this concept. A better description of “activities as usual” should be stating in the manuscript.

I will be more than happy to review the manuscript again after changes and clarifications.

VERSION 1 - AUTHOR RESPONSE

Reviewer: 1
Dr. Orfhlaith Ni Bhriain, University of Limerick
Comments to the Author:

Very thorough, well constructed paper. I appreciate the extent to which it is championed by People with PD and welcome the co-design element.

We thank the reviewer for their careful review of the manuscript and recognition of the importance of centring people with PD in the design of the trial.

Has consideration been given to participants with hearing devices?

No, we have not specifically considered the inclusion of people with hearing devices in the design of the trial, although we have kept the design deliberately broad so as not to inadvertently exclude people from participating. As we already include the line “Participants are eligible for the trial if they meet all of the following inclusion criteria and none of the exclusion criteria which have been defined to enable broad inclusivity of PwP”, we have not made any changes to the manuscript in response to this comment.

Are partners, carers welcome to attend the sessions?

Yes, partners and supporters of people with PD who are members of the group are welcome to attend the sessions as independent members. However, as we do not obtain informed consent from the supporters or partners of people included in the trial, we do not specifically collect data about this. We have added a line in the intervention section which says:

"Whilst the sessions are designed specifically for PwP, supporters of PwP or partners of PwP are welcome to engage in the group sessions of they so wish."

Will previous music/ dance experience of participants be considered?

Yes, people with previous experience of music and/ or dance are considered. We do not specifically exclude those with previous music or dance experience and they are able to join the trial as participants. We have added a short clarifying sentence to the exclusion criterion to amplify this as follows:

"The sole exclusion criterion is previous and/or present participation (self-reported) in a Sparky Samba group (this does not exclude people with prior experience of music or dance)."

P 23 Typo LINE 15 lot/Lost
P 37 Typo line 36 Participants

These typos appear in the main study protocol and not in the manuscript under review. At this time we have not changed these typos.

Reviewer: 2
Prof. Enrique Urrea Mendoza, Florida State University
Comments to the Author:
Thank you for the opportunity to review the paper entitled "A multi-centre randomize controlled feasibility trial with embedded process evaluation of a Samba percussion intervention for people living with Parkinson's disease: a protocol for the Sparky Samba trial". This is an interesting topic and the manuscript is updated and well writing. However, in order to consider publication I want clarification about enrollment and the intervention dates. It seems like the final enrollment was done on February 2026 and at this time the researchers should be in the phase of analyzing data. Per guidelines this journal can't publish protocols if data collection is completed.

We can confirm that this manuscript was submitted prior to the closure of recruitment, in line with the editorial policy for submission of protocol papers at this journal. We can further confirm that despite recruitment now being complete, the trial is still in follow up with data collection still ongoing and data analysis has not commenced.

Additionally, because this is a new intervention, a better description of the methodology, including description of instruments and graphics, will be useful for the readers that are not familiar with this concept. A better description of "activities as usual" should be stating in the manuscript.

We thank the reviewer for the comment. Whilst Sparky Samba is an uncomplicated intervention, we have provided some additional detail about the instruments available and the set-up of the group in the intervention description. We have also included a Figure to illustrate the range of instruments that may be available in a Sparky Samba session.

"In their first session participants will be offered a choice of instrument that is suitable for their range of motion. This could range across tamborims, Snare drums (Caixa), Agogo bells, Surdos, Ganzás/Chocalho (shakers), Cuíca, Timbal, Pandeiro, and the Repinique (Figure 1). The Repinique, Timbal and Surdo drums are usually played in a standing position, whereas the Tamborim, smaller Surdo drums, Caixa, Agogo bells, Ganzás/Chocalho and Pandeiro can be played in a seated position. The Repinique is carried by the player using a strap over the shoulder which suspends the drum just below the drummer's waist. Most instruments require at least one stick to play excepting the Ganzá/Chocalho or Pandeiro which require movement of only one upper limb to play. During each session, participants will be instructed to follow a rhythmic sequence, as guided by the facilitator. Typically, the room is arranged with participants in a semi-circle, grouped by instrument type, facing the facilitator. Each rhythm is broken down into smaller sections with sub-groups (grouped by instrument) concentrating on their own section before coming together. The band leader/ facilitator often carries a Repinique, as well as using Apitos (whistle) to signal breaks to participants. Each session lasts for approximately 60 minutes with time either side of the session for the set-up and break-down of instruments and seating."

Commented [PP1]: Is the band leader the same person as the facilitator? If so, this could be stated more explicitly.

With regards to the activities as usual, we have not provided a description of these as we do not know what they are specifically. Indeed, as part of our feasibility assessments we are recording what participants assigned to 'activities as usual' as we suspect these will be very varied across regions. We have provided some additional detail in the activities as usual section as follows:

"From the input of the PPI group, we anticipate that activities as usual may consist of activities such as walking, the gym, seated bowls or less physically orientated activities such as a Parkinson's choir. Given the varied availability of specific Parkinson's groups or organised community groups, we have not specified activities as usual, but will be asking people randomised to this group to specify the activities they have taken part in

over the 12 week trial period. This will help us determine the feasibility of using 'activities as usual' as a comparator condition in a future effectiveness trial of Sparky Samba."

VERSION 2 - REVIEW

**Reviewer**	**2**
**Name**	**Urrea Mendoza, Enrique**
**Affiliation**	**Florida State University**
**Date**	**24-Aug-2026**
**COI**	

Thank you very much for addressing my initial questions. I appreciate the time you took to address my comments. Your answers are clear, and I am satisfied with all your clarifications. The changes have made the paper more readable and comprehensible. Congratulations on this great work!
